# Latency of auditory evoked potential monitoring the effects of general anesthetics on nerve fibers and synapses

**DOI:** 10.1038/srep12730

**Published:** 2015-08-06

**Authors:** Bowan Huang, Feixue Liang, Lei Zhong, Minlin Lin, Juan Yang, Linqing Yan, Jinfan Xiao, Zhongju Xiao

**Affiliations:** 1 Department of Physiology, School of Basic Medical Sciences, Southern Medical University, Guangzhou 510515, PR China; 2 Department of Anesthesiology, Nanfang Hospital, Southern Medical University, Guangzhou 510515, PR China

## Abstract

Auditory evoked potential (AEP) is an effective index for the effects of general anesthetics. However, it’s unknown if AEP can differentiate the effects of general anesthetics on nerve fibers and synapses. Presently, we investigated AEP latency and amplitude changes to different acoustic intensities during pentobarbital anesthesia. Latency more regularly changed than amplitude during anesthesia. AEP Latency monotonically decreased with acoustic intensity increase (i.e., latency-intensity curve) and could be fitted to an exponential decay equation, which showed two components, the theoretical minimum latency and stimulus-dependent delay. From the latency-intensity curves, the changes of these two components (∆*L* and ∆*I*) were extracted during anesthesia. ∆*L* and ∆*I* monitored the effect of pentobarbital on nerve fibers and synapses. Pentobarbital can induce anesthesia, and two side effects, hypoxemia and hypothermia. The hypoxemia was not related with ∆*L* and ∆*I*. However, ∆*L* was changed by the hypothermia, whereas ∆*I* was changed by the hypothermia and anesthesia. Therefore, we conclude that, AEP latency is superior to amplitude for the effects of general anesthetics, ∆*L* monitors the effect of hypothermia on nerve fibers, and ∆*I* monitors a combined effect of anesthesia and hypothermia on synapses. When eliminating the temperature factor, ∆*I* monitors the anesthesia effect on synapses.

Precisely estimating the effects of general anesthetics is advantageous to decreasing the incidence of intra-operative awareness^1^, maintaining the hemodynamics more stable, and reducing the requirements for intra-operative general anesthetics, fluids and vasopressors^2^. It is necessary to continually estimate the effects of general anesthetics during anesthesia to avoid excessively deep or light anesthesia. Because auditory evoked potential is easy to be recorded and sensitive to general anesthetics^3^,^4^, it has been widely used as a measure of the effects of general anesthetics^5^,^6^. Generally, AEP is characterized by its latency (the time from stimulus beginning to the AEP wave peak) and amplitude (the voltage of the wave peak). AEP latency monotonically increases in a concentration-dependent manner during anesthesia^7^,^8^,^9^,^10^,^11^,^12^,^13^, while AEP amplitude can decrease^7^,^8^,^9^,^10^,^11^,^12^,^13^, increase^14^ or change in multiple manners during anesthesia^15^. These results suggest that AEP latency is superior to amplitude for reflecting the effects of general anesthetics. It is well known that general anesthetics have effects on both nerve fibers^16^,^17^,^18^ and synapses^16^,^19^,^20^. However, it is unknown whether or not the AEP latency change during anesthesia can reflect the effects of general anesthetics on nerve fibers and synapses.

Although some auditory neurons exhibited paradoxical latency shifts (i.e., the spike latency increases with acoustic intensity increase) in bats^21^,^22^ or a much smaller/independent latency change with acoustic intensity in bats and gerbils^23^,^24^,^25^, the first spike latency decreases as the acoustic intensity increases (i.e., the latency-intensity curve) in the inferior colliculus (IC) of mice and cats^26^,^27^. This latency-intensity curve can be fitted with an exponential decay equation, and two components—constant transmission delay (the theoretical minimum latency) and stimulus-dependent delay—can be obtained^26^,^27^,^28^,^29^. The theoretical minimum latency is regarded as the fixed travel time of sound signals through one medium, e.g., air, liquid, nerve fibers, and synaptic clefts^26^,^27^,^28^,^29^. The stimulus-dependent delay is regarded as the travel time of sound signals between two different media, e.g., presynaptic neurotransmitter release and postsynaptic membrane response time^26^,^27^,^28^,^29^.

Transduction of sound signal in air, liquid is constant, and diffusion of a given neurotransmitter in synaptic cleft is also constant. Thus, the delays in air, liquid, and synaptic cleft do not change. If the nerve fibers and synapses connected to a recording site for acoustic information processing are simplified as a functional single “wire” (as the total length of nerve fibers) and “joint” (as the summed size/strength of synapses), the theoretical minimum latency and stimulus-dependent latency can respectively reflect the propagation efficacy of sound signals (i.e., acoustic evoked spikes) in “wires” and “joints”^29^. Recently, we have developed a method to extract changes in the theoretical minimum and stimulus-dependent components of latency^29^. In this method, a double coordinate axis shift fit (DCASF) to first spike latency-intensity curves have been adopted to obtain latency and intensity shifts (∆*L* and ∆*I,* respectively), which represent the changes in the theoretical minimum and stimulus-dependent latencies, respectively^29^. Therefore, the ∆*L* and ∆*I* can monitor the changes in nerve fibers and synapses, respectively.

AEP is summary of the auditory pathways’ acoustic evoked electrical activities^30^, which based on neural spike passing along auditory pathways. The AEP latency should decrease with acoustic intensity increase as the first spike latency^26^,^27^. From the AEP latency-intensity curve, the changes in the theoretical minimum and stimulus-dependent components of AEP latency should be extracted. So, our study aimed to the changes in parameters of AEP latency that best reflects the functional changes in nerve fibers and synapses during anesthesia. In neural system, nerve fibers and synapses are two different parts in structure. Therefore, we hypothesize that the effects of general anesthetics on AEP latencies in nerve fibers and synapses may be different, that is, the changes in AEP latencies in nerve fibers and synapses can respectively reflect the different effects of general anesthetics on nerve fibers and synapses.

In this study, we first compared the variability of changes in AEP latency and amplitude recorded in the central nucleus of the inferior colliculus (ICC) in mice to different acoustic intensities during anesthesia. Then, to test our hypothesis, we attempted to extract changes in latencies in nerve fibers and synapses (∆*L* and ∆*I*) from the AEP latency during anesthesia by using DCASF^29^. In this manner we attempted to identify the effects of general anesthetics on nerve fibers and synapses.

## Results

Data were collected from forty-one mice after pentobarbital injection 90–210 min. AEP was recorded in the ICC of twenty-five mice, body temperature was measured in recta of sixteen mice, and blood oxygen saturation (SPO_2_) was monitored in the precordia of ten mice. For AEP recording, the electrodes were penetrated at 434–1266 μm from the ICC surface (Anteroposterior = −5.02 mm and mediolateral = 1.13 mm from bregma). Each recording site was characterized with its best frequency (BF).

**Figure 1:**
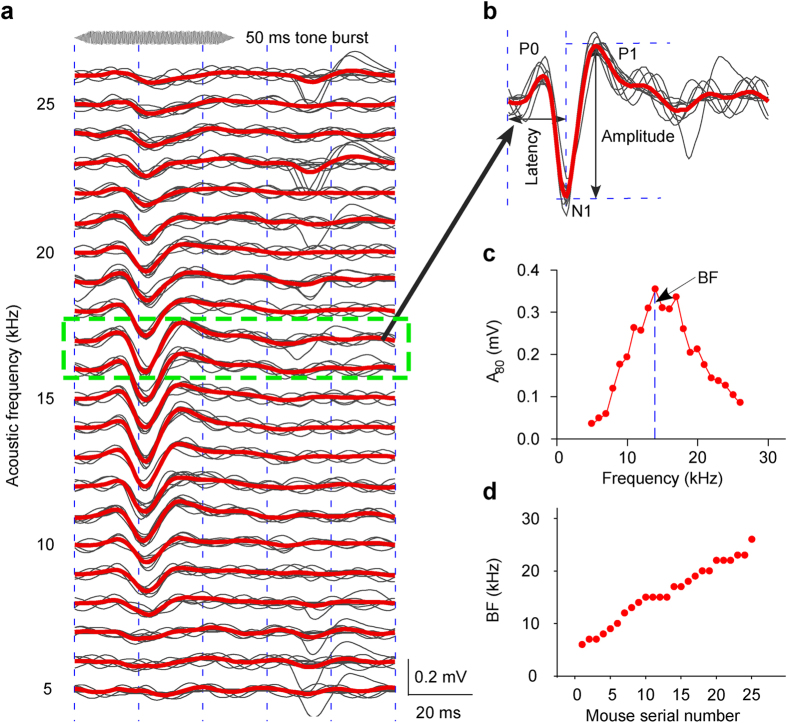
Best frequencies of recording sites. (**a**) 80 dB pure tone bursts at 5–26 kHz evoked AEPs from a recording site. (**b**) An amplificatory sample shown in a 17 kHz panel to demonstrate AEP waveforms and the extracted parameters, i.e., latency and amplitude. The AEP (red line) was average line for 10 samples (gray lines) to the same acoustic stimulus. (**c**) Definition of BF. (**d**) Summary of best frequencies of all recording sites in twenty-five mice.

To identify the BF of a recording site, pure tone bursts of various frequencies at 80 dB SPL were delivered to the mice. For example, the AEP recorded with an electrode penetrated at 816 μm depth in ICC of a mouse M20110525, tuned to 5–26 kHz pure tone bursts (Fig. 1a). At each frequency, ten AEP waveforms (Fig. 1a, the gray lines) were averaged (Fig. 1a, the red line). Each average AEP waveform had P0, N1 and P1 waves (Fig. 1b, the red line). The time from the beginning of the acoustic stimulus to the N1 peak was defined as latency, and the voltage difference from the N1 peak to the P1 peak was defined as amplitude (Fig. 1b). From 5 to 26 kHz, the amplitudes to 80 dB acoustic stimuli (A_80_) first increased to a vertex at 14 kHz, and then decreased gradually (Fig. 1c). The frequency with the largest A_80_ was defined as BF. The mice were sorted by BF of the recording site. Mouse M20110525 is No. 9 with BF 14 kHz. The best frequencies of recording sites of twenty-five mice were 6–26 kHz (Fig. 1d), which were distributed in different locations of ICC. To obtain AEP latency and amplitude to different acoustic intensities, auditory evoked potentials to seven different acoustic intensities (from 30 to 90 dB in 10 dB steps) at BF in each recording session were recorded.

### Changes in AEP latency to 80 dB acoustic stimulus (L_80_) and A_80_ during anesthesia

**Figure 2:**
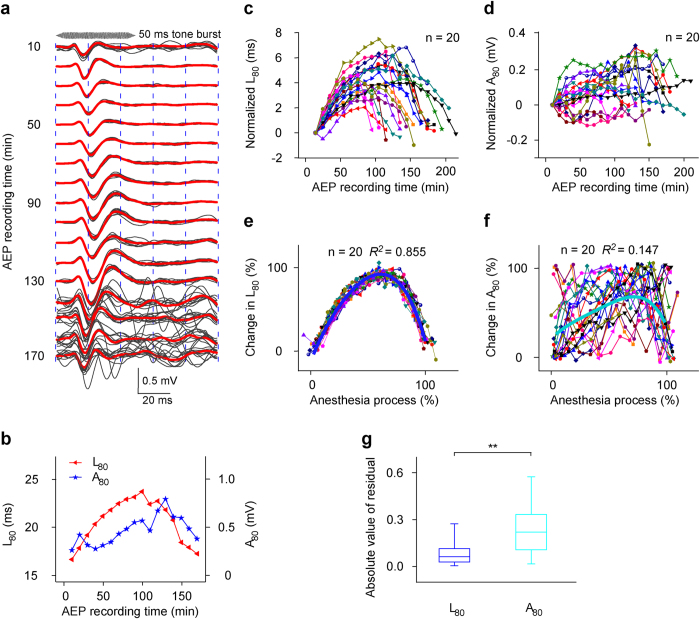
Changes in AEP to 80 dB acoustic stimuli. (**a**) The records of AEP sampled in 17 sessions lasting 170 min from M20110408 (the acoustic stimuli frequency is 12 kHz.). (**b**) The L_80_ and A_80_ changes extracted from the data shown in Fig. 2a during anesthesia were hereafter labeled as L_80_- and A_80_-time curves. (**c**,**d**) Summary of L_80_ and A_80_ changes (n = 20 mice). (**e**,**f**) The normalized L_80_- and A_80_-time curves of all mice (n = 20) and the fitting curves to a polynomial regression equation (polynomial order = 4) (blue (*R*^2^ = 0.855, intercept = −0.205, B1 = 3.789, B2 = −4.334, B3 = 3.729, B4 = −2.853) and cyan curves (*R*^2^ = 0.147, intercept = 0.214, B1 = 1.310, B2 = −2.582, B3 = 4.531, B4 = −3.073)). (**g**) Comparison between two groups of absolute values of residuals obtained by fitting normalized L_80_- and A_80_-time curves (*P*_25_ = 0.029, *P*_50_ = 0.064, *P*_75_ = 0.117, mean = 0.089 and SD = 0.083 for L_80_; *P*_25_ = 0.107, *P*_50_ = 0.221, *P*_75_ = 0.334, mean = 0.237 and SD = 0.158 for A_80_; ^**^P < 0.01, 2 Independent Samples Tests).

Because acoustic stimulus at 80 dB is usually used to collect AEP to reflect the effects of general anesthetics in the clinic^31^,^32^,^33^, we first checked the changes in AEP to 80 dB acoustic stimulus at BF of the recording site during anesthesia. A representative data from the mouse (No. 7, M20110408) to 80 dB acoustic stimuli at BF (12 kHz) showed a total of 17 AEP recording sessions lasted for 170 min after pentobarbital injection (Fig. 2a). In each recording session, ten AEP waveforms (Fig. 2a, the gray lines) were also averaged (Fig. 2a, the red line). Then, the average AEP waveform was used to measure AEP L_80_ and A_80_. During anesthesia, the L_80_ increased and then decreased (Fig. 2b, the red line with triangles to the left ordinate), whereas the A_80_ first decreased to a plateau and then increased to a maximum and then decreased with some fluctuation (Fig. 2b, the blue line with stars to the right ordinate).

The L_80_ changes (normalized by subtracting the L_80_ of the first recording session from the original L_80_ of all recording sessions) were different from mouse to mouse (Fig. 2c). However, the patterns of change were similar, namely, the L_80_ from each mouse first increased and then decreased during anesthesia. The maximum changes of the L_80_ (the peak L_80_−the first L_80_) ranged from 2.021 ms (Fig. 2c, the red line with triangles from the No. 9 mouse (M20110525)) to 7.459 ms (Fig. 2c, the dark yellow line with triangles from the No. 13 mouse (M20110413)), and occurred at 50–140 min after pentobarbital injection. The change in A_80_ (normalized by subtracting the A_80_ of the first recording session from the original A_80_ of all recording sessions) of all mice varied in different patterns (Fig. 2d).

To determine whether the differences in L_80_ or A_80_ change with anesthesia time from mouse to mouse (Fig. 2c,d) were due to the individual discrepancy of subjects, and to determine whether there was a difference between the L_80_ and A_80_ changes with anesthesia time, we normalized the whole anesthesia time as 100%, the minimums of L_80_ and A_80_ as 0%, and the maximums of L_80_ and A_80_ as 100%. When we re-plotted all the data, the normalized L_80_-time curves (Fig. 2e) basically overlapped each other and could be well fitted with a polynomial regression equation (polynomial order = 4) (Fig. 2e, the blue curve, *R*^*2*^ = 0.855). In contrast, the normalized A_80_-time curves (Fig. 2f) were not overlapping and were poorly fitted with a polynomial regression equation (polynomial order = 4) (Fig. 2f, the cyan curve, *R*^*2*^ = 0.147). The fitting resulted in the residual of each data. The residual, i.e., the difference between the actual value and the regression predicted value, can reflect the degree of variation of each data point. The absolute values of residuals obtained by fitting normalized A_80_-time curves were larger than those obtained by fitting normalized L_80_-time curves (Fig. 2g, 2 Independent Samples Tests, *Z* = −12.519, *P* = 5.890 × 10^−36^). Therefore, the AEP L_80_ from all mice changed with more regularity and less variability than the AEP A_80_ during pentobarbital anesthesia.

### Changes in AEP latency and amplitude to various acoustic intensities during anesthesia

**Figure 3:**
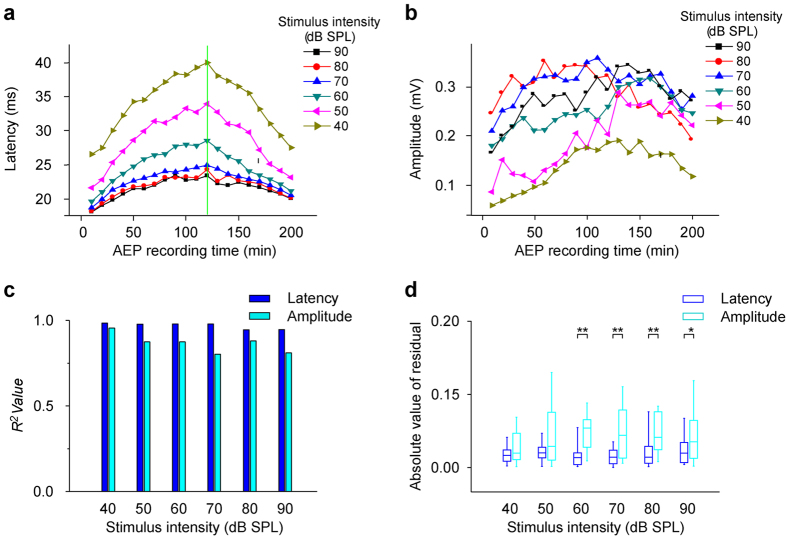
Changes in AEP latency and amplitude to different acoustic intensities. (**a**,**b**) Changes in latency and amplitude to 40–90 dB acoustic stimuli with anesthesia time, hereafter, latency- and amplitude-time curves. (c and d) *R*^2^ values and absolute values of residuals obtained by fitting normalized latency- and amplitude-time curves (computing methods were similar to those in Fig. 2) (^*^P < 0.05, ^**^P < 0.01, 2 Independent Samples Tests).

Although AEP L_80_ exhibited more regular and less variable changes than A_80_, whether AEPs to acoustic stimuli at other intensities undergo similar changes during anesthesia is still unclear. Therefore, we applied multiple acoustic intensities other than 80 dB to the subjects. The latency to each acoustic intensity (No. 18 mouse (M20110417)) showed an initial increase followed by a gradual decrease during anesthesia (Fig. 3a). The maximal latency to each acoustic intensity always occurred in the same recording session (Fig. 3a, the vertical green line). The latency change ranges (the maximal latency—the minimal latency) to different acoustic intensities were, respectively, 13.475, 12.344, 8.935, 6.23, 6.147 and 5.41 ms from 40 dB to 90 dB at 10 dB intervals, and were negatively correlated with the acoustic intensity. These results indicated that AEP latencies to low intensity acoustic stimuli were more suitable for reflecting the effects of general anesthetics.

Although the amplitude (No. 18 mouse (M20110417)) also increased and then decreased during anesthesia to a given intensity acoustic stimulus, less regularity was found (Fig. 3b). Generally, the amplitude change to higher acoustic intensity was at a higher level (Fig. 3b. the curves for 40–80 dB). However, the amplitude change to a 90 dB acoustic stimulus (Fig. 3b, the black line with squares) decreased paradoxically when compared with those to 70 and 80 dB (Fig. 3b, the blue line with triangles and red line with circles). The maximal amplitudes to different acoustic intensities occurred in different recording sessions (Fig. 3b). Furthermore, the amplitude change ranges (the maximal amplitude—the minimal amplitude) to different acoustic intensities were, respectively, 0.132, 0.202, 0.137, 0.148, 0.16 and 0.178 mV from 40 dB to 90 dB at 10 dB intervals, and showed little regular change with acoustic intensity increase.

When we applied the same methods as those in Fig. 2e to latency- and amplitude-time curves in Fig. 3a,b, at any acoustic intensity, the *R*^2^ value of fitting the normalized latency-time curve was larger than that of fitting the normalized amplitude-time curve (Fig. 3c). The fitting *R*^2^ values at low intensity acoustic stimuli (40, 50, 60 and 70 dB in latency; 40 dB in amplitude) were larger than those at high-intensity acoustic stimuli (80 and 90 dB in latency; 50, 60, 70, 80 and 90 dB in amplitude) (Fig. 3c). Additionally, at any acoustic intensity, the absolute values of residuals of fitting normalized latency-time curves were smaller than those of fitting the normalized amplitude-time curve, although at 40 and 50 dB, there was no statistically significant difference between two absolute values of residuals (Fig. 3d, 2 Independent Samples Tests). For latency, the absolute values of residuals of fitting at various intensity acoustic stimuli were similar to each other (Fig. 3d, the blue column). For amplitude, the absolute values of residuals of fitting at low intensity acoustic stimuli (40 and 50 dB) were smaller than those at high intensity acoustic stimuli (60, 70, 80 and 90 dB) (Fig. 3d, the cyan column).

### Extracting changes in nerve fibers and synapses from AEP latencies during anesthesia

**Figure 4:**
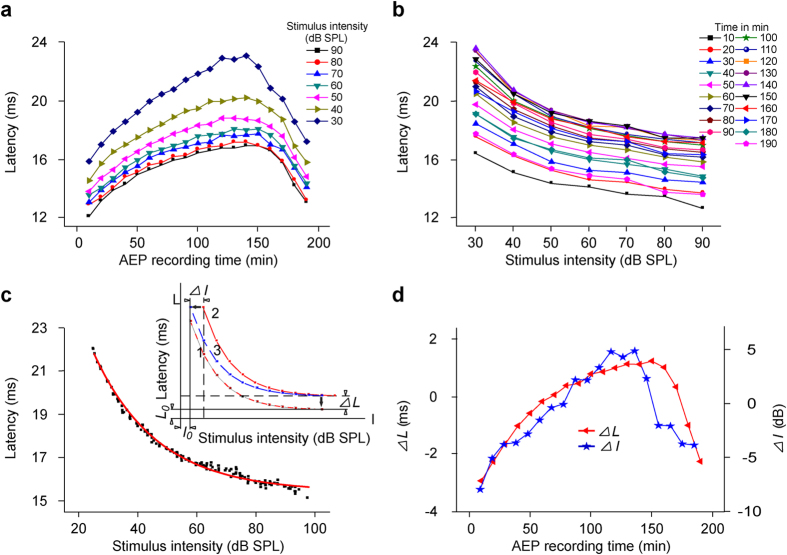
One example of DCASF. (**a,b**) Latency-time curves to 30–90 dB acoustic stimuli and latency changes with acoustic intensity for all recording sessions lasting 190 min, hereafter, latency-intensity curves (**c**) Data fitting to DCASF. The average latency-intensity curve of all latency-intensity curves in Fig. 4b was fit to Equation (1) (the red line was the fitting curve, i.e., reference curve (corresponding to Curve 1 in the insert)). Then, each curve in Fig. 4b as the target curve (corresponding to Curve 2 in the insert) was fit to Equation (2) to obtain ∆*L* and ∆*I* (insert). This result can be simplified as the target curve (Curve 2) first shifts ∆*I* to overlap with Curve 3 and then shifts ∆*L* to overlap with the reference curve (Curve 1) along the axes (the insert) (details introduced in Results). The latency shift (∆*L*) and intensity shift (∆*I*) reflect the changes of latencies in nerve fibers and synapses, respectively. When each data point in Fig. 4b subtracted its corresponding shifts (∆*L* and ∆*I*) and those newly obtained data were re-plotted, the black dots were obtained. (**d**) ∆*L* and ∆*I* changes with anesthesia time were hereafter labeled as ∆*L*- and ∆*I*-time curves.

Recently, we have developed a method (DCASF) to first spike latency-intensity curves recorded in single cells, to extract changes in the theoretical minimum and stimulus-dependent components of latency^29^, which can represent the changes in spike latencies in nerve fibers and synapses, respectively. The latency changes of AEPs with anesthesia time (latency-time curves) from No. 5 mouse (M20110410) (Fig. 4a) were similar to those shown in Fig. 3a. When the data were re-plotted in latency-intensity curve for each recording session (Fig. 4b), the latency exponentially decayed as acoustic intensity increased, as previous results from single cells^26^,^27^, although AEP is summary of the acoustic evoked electrical activities of auditory pathways^30^. Thus, we can analyze the effects of general anesthetics on nerve fibers and synapses by using DCASF^29^.

To perform DCASF, a latency-intensity curve was fit to Equation (1) first:


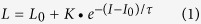


where *L* and *I* represent AEP latency and the corresponding acoustic intensity, respectively. *L*_0_, *I*_0_, *K*, and *τ* are four constants from the fitting. *L*_0_ is the theoretical minimum latency, i.e., the asymptotic latency, with which *L* converges as the acoustic intensity (or the magnitude of X abscissa) approaches infinity. This variable includes the acoustic delay, middle ear and cochlear delays, fixed delays of the synapse, and the axonal travel time of spikes to the recording site^26^. This constant delay is referred to as the transmission delay^27^,^28^,^29^. *I*_0_ is the theoretical minimum intensity of the acoustic stimulus for an infinite time, i.e., asymptotic intensity, with which *I* converges as latency (or the magnitude of Y ordinate) approaches infinity. *K* is the coefficient of delay for a stimulus. *τ* is an exponential decay factor.

The fit curve determined by the four constants*—L*_0_, *I*_0_, *K*, and *τ* (Fig. 4c, the insert Curve 1) was taken as a reference. Then, a target curve (Fig. 4c, the insert Curve 2) was fit to Equation (2):


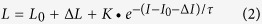


where ∆*I* and ∆*L* are the shifts on *I* abscissa and *L* ordinate, respectively, for the target curve (Fig. 4c, the insert Curve 2) to overlap with the reference curve. The four constants*—L*_0_, *I*_0_, *K*, and *τ* are from the fit to Equation (1).

The average latency-intensity curve of all latency-intensity curves (Fig. 4b) from the subject was closely fit to Equation (1) (*R*^2^ = 0.997; *L*_0_, *I*_0_, *K* and *τ* were 15.426, 29.705, 5.160 and 21.819, respectively). With the fitting curve (Fig. 4c, the red curve) as the reference, i.e., based on the fit constants, the latency-intensity curve for each recording session (Fig. 4b) was fit to Equation (2) in order to obtain its latency shift (∆*L*) and intensity shift (∆*I*) (Fig. 4d). ∆*L* and ∆*I* reflected the changes in the theoretical minimum latency (*L*_0_) and stimulus-dependent delay (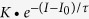), respectively^29^. When each dot on the latency-intensity curves subtracted its corresponding shifts (∆*I*, ∆*L*), the superposition of all latency-intensity curves of this mouse (Fig. 4c, the black dots) were obtained. Then, we fitted this superposition with Equation (2) and obtained the ∆*L*, ∆*I* and *R*^2^ values, which, respectively, were 0.0006, –0.0059 and 0.991. The tiny shifts and high *R*^2^ value meant that all latency-intensity curves could be highly superimposed on the reference curve by subtracting the corresponding shifts (Fig. 4c, the black dots). During anesthesia, the ∆*L* (Fig. 4d, the red line with triangles to the left ordinate) and ∆*I* (Fig. 2b, the blue line with stars to the right ordinate) first increased and then decreased; however, the ∆*L* changed more smoothly than the ∆*I*.

### The effects of pentobarbital anesthesia on nerve fibers and synapses read by ∆*L* and ∆*I* from AEP latency-intensity curves

**Figure 5:**
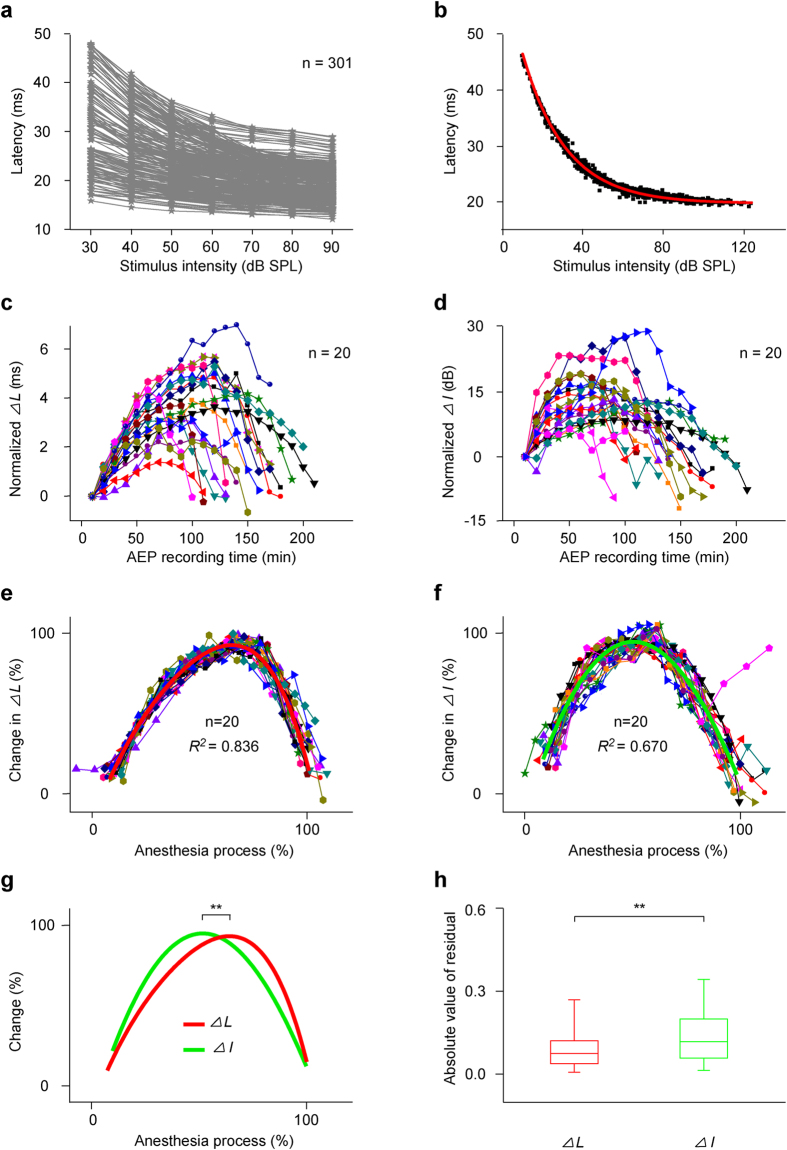
Changes in ∆*L* and ∆*I*. (**a**) Latency-intensity curves (n = 301) for each recording session of twenty mice. (**b**) Data fitting to DCASF. Data were presented as those in Fig. 4c. (**c**,**d**) Summary of ∆*L*- and ∆*I*-time curves (n = 20). (**e,f**) The normalized ∆*L*- and ∆*I*-time curves of twenty mice and the fitting curves to a polynomial regression equation (polynomial order = 4) (red (*R*^2^ = 0.836, intercept = −0.225, B1 = 4.036, B2 = −6.073, B3 = 7.034, B4 = −4.622) and green curves (*R*^2^ = 0.670, intercept = −0.020, B1 = 3.822, B2 = −3.821, B3 = −0.269, B4 = 0.410)). Normalized and fitting methods were similar to those in Fig. 2e. (**g**) Comparison between the two fitting curves (peak time: 0.688 ± 0.103 vs. 0.547 ± 0.115, ^**^P < 0.01, unpaired *t* test). (**h**) Comparison between two groups of absolute values of residuals obtained by fitting normalized ∆*L*- and ∆*I*-time curves (*P*_25_ = 0.038, *P*_50_ = 0.074, *P*_75_ = 0.122, mean = 0.096 and SD = 0.087 for ∆*L*; *P*_25_ = 0.058, *P*_50_ = 0.118, *P*_75_ = 0.202, mean = 0.141 and SD = 0.112 for ∆*I*; ^**^P < 0.01, 2 Independent Samples Tests).

The latency-intensity curves of all recording sessions from twenty sampled mice had similar shapes and showed an exponential decay relationship between latency and acoustic intensity (Fig. 5a, n = 301). The average curve of all latency-intensity curves in Fig. 5a was also fit well to Equation (1) (*R*^2^ = 0.998; *L*_0_, *I*_0_, *K* and *τ* were 19.607, 29.754, 10.984 and 21.443, respectively). Taking the fitting curve (Fig. 5b, the red curve) as the reference curve, the corresponding shifts (∆*L* and ∆*I*) for each latency-intensity curve were obtained by performing DCASF on all latency-intensity curves in Fig. 5a. After subtracting the corresponding ∆*L* and ∆*I*, all latency-intensity curves overlapped well with the reference curve (Fig. 5b, the black dots), which were well fit to Equation (2). The ∆*L*, ∆*I* and *R*^2^ values were 0.00016, –0.00021 and 0.998, respectively. That is, the fitting curve almost was the same as the reference curve. To show the shifts (∆*L* and ∆*I*) during anesthesia in each mouse, ∆*L* and ∆*I* for the first recording session were referred to zero, and all other shifts were normalized to those and plotted with recording session, i.e., time (Fig. 5c,d).

For all mice, although the changes in ∆*L* (Fig. 5c) or ∆*I* (Fig. 5d) with anesthesia time were different from mouse to mouse, the change regularity of ∆*L* or ∆*I* was similar. ∆*L* or ∆*I* increased and then decreased with anesthesia time. The maximum changes of ∆*L* (the peak ∆*L*—initial ∆*L*) ranged from 1.382 ms (Fig. 5c, the red line with triangles from the No. 9 mouse M20110525) to 7.015 ms (Fig. 5c, the royal blue line with circles from the No. 11 mouse M20110501) and occurred at 50–150 min after pentobarbital injection. The maximum changes of ∆*I* (the peak ∆*I*—initial ∆*I*) were from 6.499 dB (Fig. 5d, the magenta line with pentagons from the No. 15 mouse M20110613) to 29.284 dB (Fig. 5d, the blue line with triangles from the No. 6 mouse M20110528) and occurred at 40–120 min. The peak times for ∆*L*-time curves (106.0 ± 24.4 min) were significantly different from those for ∆*I*-time curves (82.5 ± 26.7 min) (Fig. 5c,d, unpaired *t* test, *t* (38) = 2.906, *P* = 0.006).

The changes of ∆*L* (Fig. 5c) or ∆*I* (Fig. 5d) with anesthesia time (∆*L*-time curves or ∆*I*-time curves) were similar in shape for all mice, but different from mouse to mouse. To further compare these curves, we applied the same methods as those in Fig. 2e. The normalized ∆*L* (Fig. 5e) or ∆*I* (Fig. 5f) time curves basically overlapped. Both could be well fit with a polynomial regression equation (polynomial order = 4) (Fig. 5e,f, the red and green curves). Plotting the above two fitting curves together showed that there was a clear difference between the two (Fig. 5g). The peak times for normalized ∆*L*-time curves were later than those for normalized ∆*I*-time curves (Fig. 5g, unpaired *t* test, *t* (38) = 4.102, *P* = 2.080 × 10^−4^). In addition, the absolute values of residuals obtained by fitting normalized ∆*I*-time curves were larger than those obtained by fitting normalized ∆*L*-time curves (Fig. 5h, 2 Independent Samples Tests, *Z* = −5.703, *P* = 1.170 × 10^−8^). In other words, the ∆*L* had less variability than ∆*I* and was superior to ∆*I* for reflecting the effects of general anesthetics.

### Hypoxemia and hypothermia induced by pentobarbital compared with the changes of L_80_, ∆*L* and ∆*I* during anesthesia

**Figure 6:**
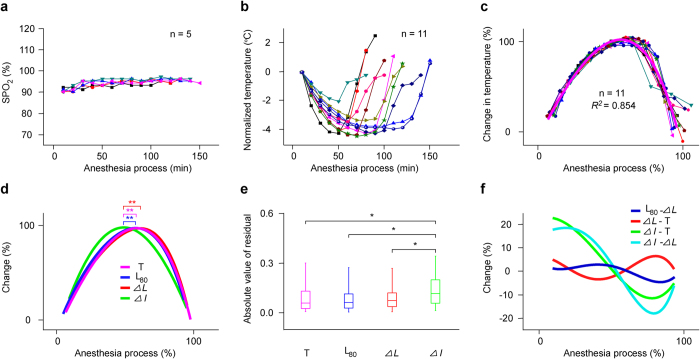
Comparison among changes in SPO_2_, temperature, L_80_, ∆*L* and ∆*I*. (**a,b**) SPO_2_ and temperature changes with anesthesia time were hereafter labeled as SPO_2_- and temperature-time curves. (**c**) The normalized temperature-time curves of mice (n = 11) and the fitting curves to a polynomial regression equation (polynomial order = 4) (magenta curve (*R*^2^ = 0.854, intercept = −0.144, B1 = 3.575, B2 = −2.122, B3 = −1.029, B4 = −0.273). The normalized and fitting methods were similar to those in Fig. 2e. (**d**) Comparison among temperature, L_80_, *∆L* and *∆I* fitting curves (peak time: 0.547 ± 0.115 (*∆I* curve) vs. 0.642 ± 0.059 (T curve), ^**^P < 0.01; peak time: 0.547 ± 0.115 (∆*I* curve) vs. 0.643 ± 0.114 (L_80_ curve), ^**^P < 0.01; peak time: 0.547 ± 0.115 (*∆I* curve) vs. 0.688 ± 0.103 (*∆L* curve), ^**^P < 0.01; one-way ANOVA and multiple comparison LSD’s test). (**e**) Comparison among four groups of absolute values of residuals obtained by fitting normalized temperature-, latency-, *∆L*- and *∆I*-time curves (*P*_25_ = 0.026, *P*_50_ = 0.058, *P*_75_ = 0.136, mean = 0.091 and SD = 0.093 for temperature; *P*_25_ = 0.029, *P*_50_ = 0.064, *P*_75_ = 0.117, mean = 0.089 and SD = 0.083 for latency; *P*_25_ = 0.038, *P*_50_ = 0.074, *P*_75_ = 0.122. mean = 0.096 and SD = 0.087 for ∆*L*; *P*_25_ = 0.058, *P*_50_ = 0.118, *P*_75_ = 0.202, mean = 0.141 and SD = 0.112 for ∆*I*; ^*^P < 0.008, K Independent Samples Tests and multiple comparison 2 Independent Samples Tests). (**f**) Curves after one fitting curve (L_80_, ∆*L* or ∆*I*) in Fig. 6C subtracted another fitting curve (∆*L* or T) in Fig. 6C.

Pentobarbital can induce hypoxemia^34^ and hypothermia^35^. Therefore, the SPO_2_ and rectal temperature (T) were measured every 10 min after pentobarbital anesthesia. The SPO_2_ always decreased at initial 20 min or 30 min, then increased and kept relatively steady during anesthesia (Fig. 6a). The SPO_2_ were not lower than 90%. The SPO_2_ changes were not similar to the changes of L_80_ (Fig. 2c), ∆*L* (Fig. 5c) and ∆*I* (Fig. 5d) in shape. However, temperature changes, normalized by subtracting the temperature of the first recording time point from the original temperatures of all recording time points, were similar in shape for all mice (Fig. 6b), which seemed inversely to the changes of the L_80_ (Fig. 2c), ∆*L* (Fig. 5c) and ∆*I* (Fig. 5d). The normalized temperature-time curves (Fig. 6c) with the same methods as those in Fig. 2e overlapped well and could be fit with a polynomial regression equation (polynomial order = 4) (Fig. 6c, the magenta curve). Plotting the fitting curves for the T-, L_80_-, ∆*L-* and ∆*I-*time curves together (Fig. 6d), shows that the three curves for the T, L_80_ and ∆*L* were almost superimposed with no significant difference in the peak time between any two curves (one-way ANOVA, *F* (3, 67) = 6.454, *P* = 0.001; multiple comparison LSD’s test, *P* = 0.976 for T and L_80_, *P* = 0.240 for T and ∆*L*, *P* = 0.174 for L_80_ and ∆*L*), but that a clear difference between the ∆*I* fitting curve and the T, L_80_ or ∆*L* fitting curve (one-way ANOVA, *F* (3, 67) = 6.454, *P* = 0.001; multiple comparison LSD’s test, *P* = 0.018 for ∆*I* and T, *P* = 0.005 for ∆*I* and L_80_, *P* = 5.979 × 10^−5^ for ∆*I* and ∆*L*). In addition, the absolute values of residuals obtained by fitting normalized ∆*I*-time curves were larger than any one of the other three residuals for T-, L_80_- or ∆*L*-time curves (Fig. 6e, K Independent Samples Tests, *x*^*2*^ = 58.333, *P* = 3.130 × 10^−7^, multiple comparison 2 Independent Samples Tests, *P* = 3.134 × 10^−7^ for ∆*I* and T, *P* = 2.110 × 10^−11^ for ∆*I* and L_80_, *P* = 1.174 × 10^−8^ for ∆*I* and ∆*L*), but there was no significant difference between any two of the latter three residuals (Fig. 6e, K Independent Samples Tests, *x*^*2*^ = 58.333, *P* = 3.130 × 10^−7^, multiple comparison 2 Independent Samples Tests, *P* = 0.593 for T and L_80_, *P* = 0.120 for T and ∆*L*, *P* = 0.169 for L_80_ and ∆*L*). Furtherly, the L_80_ fitting curve subtracted the ∆*L* fitting curve showed almost a line fluctuated around zero (Fig. 6f, the blue curve). It also happened to the ∆*L* fitting curve subtracted the T fitting curve (Fig. 6f, the red curve). However, the ∆*I* fitting curve subtracted the T fitting curve or the ∆*L* fitting curve presented a similar gradual decrease tendency with anesthesia time (Fig. 6f, the green or cyan curve).

### The changes of SPO_2_, T, ∆*L* and ∆*I* during anesthesia with body warming

Because the ∆*L* fitting curve subtracted the T fitting curve almost showed a line fluctuated around zero (Fig. 6f, the red curve), it seemed that ∆*L* change might be due to the hypothermia induced by pentobarbital. Therefore, with rectal temperature and SPO_2_ monitoring, we kept the temperatures of mice steady during pentobarbital anesthesia by a homeothermic blanket, and reexamined ∆*L* and ∆*I* changes.

**Figure 7:**
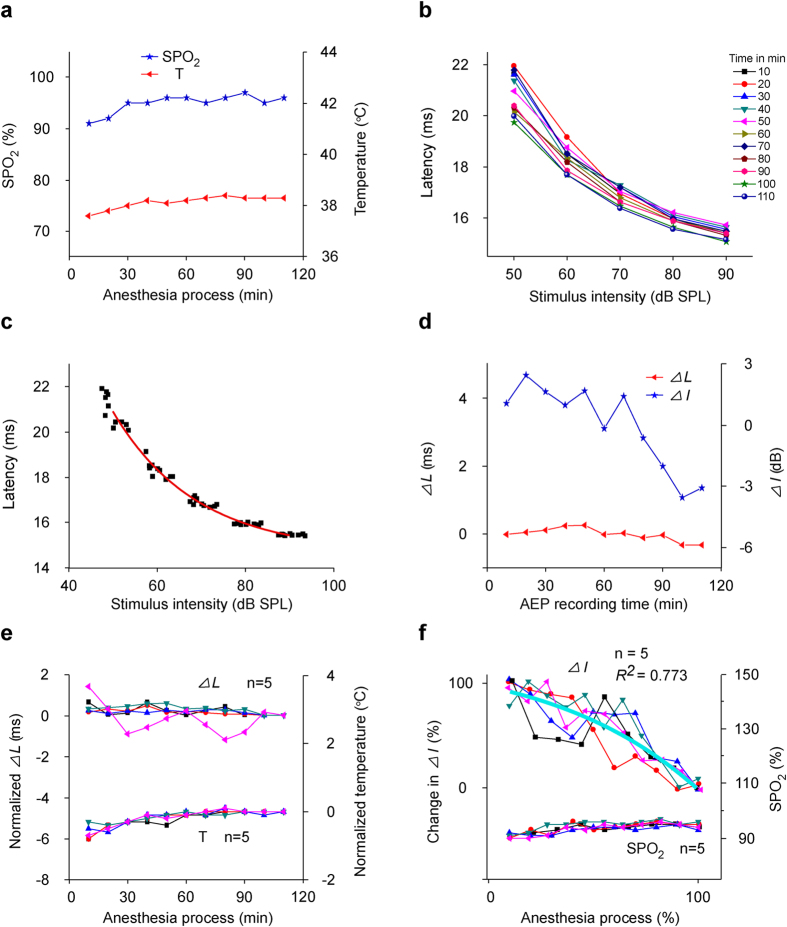
Changes in SPO_2_, temperature, ∆*L* and ∆*I* in warming mice. (**a**) SPO_2_- and temperature-time curves (M20150325). (**b**) Latency-intensity curves from M20150325 for all recording sessions lasting 110 min. (**c**) Data fitting to DCASF. Data were presented as those in Fig. 4c. (**d**) ∆L- and ∆I-time curves obtained from Fig. 7b. (**e**) ∆L- and temperature-time curves of five mice. (**f**) Normalized *∆I*-time curves of five mice and the fitting curves to GaussAmp equation (cyan curve,
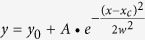
, *R*^2^ = 0.773, y_0_ = 1.208, A = −92.980, x_c_ = 7.112, w = 2.073), and SPO_2_-time curves of five mice.

The SPO_2_ of mouse (M20150325) decreased at initial 20 min, then increased and kept relatively steady during the anesthesia (Fig. 7a, the blue curve with stars to the left ordinate). The SPO_2_ were not lower than 91%. With body warming, although the temperatures measured from this mouse slightly decreased at initial 20 min after pentobarbital injection, the temperatures were normal and relative steady during recording. The fluctuation range of temperatures did not exceed 1 ºC (Fig. 7a, the red curve with triangles to the right ordinate). The AEP latency from this mouse also exponentially decayed with acoustic intensity (latency-intensity curves) (Fig. 7b), but the range of latency changes was much less than those without body warming (Figs 4b, 5a). By performing DCASF as that to the data in Figs 4c, 5b, all latency-intensity curves in Fig. 7b could be fitted to Equation (2) with *L*_0_, *I*_0_, *K* and *τ* were respectively 14.683, 47.732, 7.013 and 18.859 from the fit to Equation (1) (Fig. 7c, the red curve, *R*^2^ = 1.000), and their ∆*L* and ∆*I* during anesthesia were obtained (Fig. 7d). Not surprising, the ∆*L* kept relatively steady during anesthesia and the fluctuation range of ∆*L* did not exceed 0.6 ms (Fig. 7d, the red curve with triangles to the left ordinate). However, the ∆*I* gradually decreased as anesthesia time with some fluctuations (Fig. 7d, the blue curve with stars to the right ordinate).

The temperatures (normalized by subtracting the temperature of the first recording time point from the original temperatures of all recording time points) of five mice were kept well steady with a homeothermic blanket during anesthesia. The rectal temperatures fluctuation of each mouse was less than 1 ºC (Fig. 7e, the nether curves to the right ordinate). Correspondingly, the ∆*L* (normalized by subtracting the ∆*L* of the first recording session from the original ∆*L* of all recording sessions) showed little change during anesthesia, except in one mouse with a 1.496 ms fluctuation, but which presented a relative steady tendency (Fig. 7e, the upper curves to the left ordinate). However, the ∆*I* for all mice showed gradually decrease with anesthesia time after normalized as the data processing in Fig. 2e (Fig. 7f, the upper curves to the left ordinate). These curves could be fitted to a GaussAmp equation (Fig. 7f, the upper cyan curve), which was similar to the tendency of the ∆*I* fitting curve subtracted the T fitting curve or the ∆*L* fitting curve (Fig. 6f, the green and cyan curve), and totally different from the normalized ∆*I* curve from without warming animals (Fig. 5f). Similar to the rectal temperature, the SPO_2_ for all mice changed little (Fig. 7f, the nether curve to the right ordinate). The changes in SPO_2_ were similar to those in mice without body warming (Fig. 6a).

## Discussion

In neural system, nerve fibers and synapses are two different parts in structure. General anesthetics clearly affect nerve fibers^16^,^17^,^18^ and synapses^16^,^19^,^20^. AEP latency is suggested to be a more effective index for the effects of general anesthetics than AEP amplitude^7^,^8^,^9^,^10^,^11^,^12^,^13^,^14^,^15^. In this study, we have explored whether AEP latency can differentiate the effects of general anesthetics on nerve fibers and synapses. Firstly, we extracted and systematically compared the specialties of the two characteristic parameters, latency and amplitude of AEP during pentobarbital anesthesia. Secondly, we extracted the changes in nerve fibers and synapses, i.e., ∆*L* and ∆*I* from the AEP latency-intensity curves during pentobarbital anesthesia. Pentobarbital does not only induce anesthesia, but also has two side effects, hypothermia and hypoxemia. Then by body warming, temperature monitoring and SPO_2_ monitoring, we straightened out the relationship of ∆*L* and ∆*I* with anesthesia, hypothermia and hypoxemia. This study suggests that AEP latency is superior to amplitude for reflecting the effects of general anesthetics, ∆*L* monitors the effect of hypothermia on nerve fibers, ∆*I* monitors a combined effect of anesthesia and hypothermia on synapses.

### AEP latency superior to amplitude for reflecting the effects of general anesthetics

This study has shown that AEP latency exhibited similar changes in different subjects and at different acoustic intensities during anesthesia (Figs 2e, 3a), corroborating previous studies^36^,^37^,^38^,^39^,^40^. However, AEP amplitude changed with greater variability during anesthesia (Figs 2g, 3d), consistent with other reports^14^,^41^. Therefore, the latency parameter of AEP is superior to amplitude in accuracy and stability in response to acoustic stimuli. This finding indicates that latency serves as a better index than amplitude for reflecting the effects of general anesthetics. Although the AEP amplitude for given subjects or given acoustic intensities had some relationship with anesthesia time (Figs 2d, 3b), amplitude as a parameter of AEP should not be used to reflect the effects of general anesthetics if we consider the variability of changes in amplitude in all mice or at various acoustic intensities (Figs 2f, 3b).

Here, AEP is a local field potential that is based on the spatial and temporal superposition of acoustic evoked electrical activities of auditory pathways^30^. As the electrical activities of auditory pathways at a single time point increase, the electrical activities become increasingly spatially superimposed, leading to a larger AEP amplitude^42^. As the synchronized degree of electrical activities of auditory pathways increase, the more electrical activities become temporally superimposed at a single time point, leading to a larger AEP amplitude^42^. Although anesthesia can decrease the firing rate of neural units (that is, acoustic evoked electrical activities at one time point decrease)^43^ and decrease the spatial superposition of electrical activities, it can also increase the synchronization of electrical activities^14^ and increase the temporal superposition of electrical activities. Therefore, it does make sense that amplitude is more fickle during anesthesia (Figs 2g, 3d).

The AEP to acoustic stimulus at 80 dB is usually used to reflect the effects of general anesthetics in a clinical setting^31^,^32^,^33^. However, the AEP latency changes during anesthesia were more sensitive to low acoustic intensities than to higher acoustic intensities (Fig. 3a). In addition, at low-intensity acoustic stimuli, the fitting *R*^2^ values were larger (Fig. 3c). These results indicate that AEP latency to low intensity acoustic stimuli should be adopted to reflect the effects of general anesthetics.

### AEP latency change in nerve fibers (∆*L*) as AEP latency to high acoustic intensity (e.g., L_80_) monitoring the effect of hypothermia on nerve fibers

AEP latency change includes two component changes, latency changes in the nerve fibers (∆*L*) and synapses (∆*I*). Change in ∆*I* was different from that in ∆*L* (Fig. 6d,e). Thus, change in latency should be also different from that in ∆*L* and ∆*I*. However, in this study, change in L_80_ was almost same as that in ∆*L* during anesthesia (Fig. 6d,e). The potential reason for this pattern is that we chose to observe AEP latency to high intensity (80 dB) acoustic stimuli. As seen from the latency-intensity curve (Fig. 4c, insert), as the acoustic intensity increases, the latency more closely approximates the theoretical minimum latency (*L*_0_). Thus AEP L_80_ approximated the theoretical minimum latency (*L*_0_) (Fig. 5b). During anesthesia, the change of *L*_0_ is ∆*L*. Therefore, AEP L_80_ change was basically consistent with the ∆*L* change.

Without body warning for mice, the ∆*L* (Fig. 5c) and L_80_ (Fig. 2c) reached their maximum at tens of minutes after pentobarbital injection. However, the pentobarbital concentration in the brain after intravenous injection increases to a maximum at 4 min and then decreases slowly^44^. Although the concentration reaches a peak at a relatively longer time after intra-peritoneal injection than after intravenous injection, it is obviously shorter than tens of minutes. Thus, the changes in ∆*L* and L_80_ can not be only explained with the anesthesia effect of pentobarbital.

Pentobarbital can induce hypoxemia^34^, which can change AEP^45^. Thus, pentobarbital-induced hypoxemia might change ∆*L* and L_80_. However, the SPO_2_ were relatively steady after pentobarbital injection 30 min and not lower than 90% with or without body warming (Figs 6a, 7f), it was not severe hypoxemia during anesthesia. According to previous study, AEP latency is insensitive to non-severe hypoxemia^46^,^47^. Additionally, the changes in SPO_2_ in warming mice (Fig. 7f) were similar to those in non-warming mice (Fig. 6a), while the changes in ∆*L* and L_80_ without body warming were eliminated by body warming. Thus, the hypoxemia did not change the AEP latency.

Without body warming, the brain temperature in rats rapidly decreases at 10–20 min after intravenous sodium pentobarbital, reaches its nadir at 80–110 min, and then gradually increases^35^. Hypothermia can depress nerve fiber transmission^17^,^18^,^48^ and synaptic conduction^48^,^49^,^50^, and consequently lengthen AEP latency^51^. Thus, pentobarbital-induced hypothermia might also change ∆*L* and L_80_ in this study. This can be confirmed by that the changes in ∆*L* (Fig. 5e) without body warming were different from those with body warming (Fig. 7e).

In this study, without body warming, the changes in T, ∆*L* and L_80_ were similar to each other (Fig. 6d,e). When the body temperatures of mice were kept steady, the ∆*L* changed little (Fig. 7e). Therefore, the change in ∆*L* and L_80_ were primarily caused by pentobarbital-induced hypothermia, not by the anesthesia effect of pentobarbital (Figs 6d,e, 7e). This was supported by that nerve fiber transmission is not easily depressed by the anesthesia effect of pentobarbital under constant normal temperature^52^. In other words, ∆*L* and L_80_ can be used for monitoring the effect of hypothermia on nerve fibers during anesthesia. To reflect the anesthesia effect of pentobarbital with AEP latency, low-intensity acoustic stimuli might be adopted.

### AEP latency change in synapses (∆*I*) monitoring a combined effect of anesthesia and hypothermia on synapses

Similar to analysis in ∆*L*, change in ∆*I* was not related with pentobarbital-induced hypoxemia, and can also not be only explained with the anesthesia effect of pentobarbital. Without body warming, the change in ∆*I* was obviously different from the rectum temperature change (Fig. 6d,e) and the brain pentobarbital concentration change during anesthesia^44^. With body warming, the change in ∆*I* (Fig. 7f) was similar to the change in brain pentobarbital concentration during anesthesia^44^. Thus, ∆*I* was changed by hypothermia and the anesthesia effect of pentobarbital. That is, ∆*I* can monitor a combined effect of anesthesia and hypothermia on synapses. With body warming, ∆*I* only reflects the anesthesia effect of pentobarbital on synapses. Without body warming, if the ∆*I* data subtracted the temperature or ∆*L* data, ∆*I* might also reflect the anesthesia effect of pentobarbital on synapses (Fig. 6f). Thus, this study suggests a method to estimate the anesthesia effect of a general anesthetic with hypothermia.

In conclusion, AEP latency is superior to amplitude for the effects of general anesthetics. Latency to low intensity acoustic stimuli is superior to latency to high intensity acoustic stimuli. Latency change in nerve fibers, as latency to high-intensity acoustic stimuli, can reflect the effect of hypothermia on nerve fibers. Latency change in synapses can reflect a combined effect of anesthesia and hypothermia on synapses. When eliminating the temperature factor, latency change in synapses can reflect the anesthesia effect of pentobarbital on synapses.

## Methods

### General

The study protocols were approved by the Animal Care and Use Committee of Southern Medical University. Forty-one female BALB/c mice aged 4–6 weeks and weighing 12–18 g with normal hearing were obtained from the Experimental Animal Center of Southern Medical University, Guangzhou, China. All mice were handled according to the guidelines set by the Animal Care and Use Committee of Southern Medical University. The surgical procedures, acoustic stimulation methods, and methods for recording electrophysiological activity in this experiment were similar to those described previously^27^,^28^,^29^.

### Surgical preparation

After anesthesia with sodium pentobarbital (60–70 mg/kg, i.p.) and atropine sulfate (0.25 mg/kg, s.c., for reducing tracheal mucous secretion), each mouse’s skull was exposed under sterile conditions and a reference electrode was placed under prefrontal bone. Next, a 1.5-cm-long nail was fixed to its dorsal surface with dental cement. Then, the nail was inserted into a small metal rod, which was tightly connected to an anti-vibration table in a soundproof room (temperature maintained at 24–26 °C), and was fixed by screws to immobilize the mouse’s head. Subsequently, a 2 × 2-mm^2^ skull section on the IC was removed under a surgical microscope (WPI, USA). Then, the exposed brain was covered with vaseline plastic wrap and tissue. After that, the mouse was put back into a cage for at least two days of recovery.

### Acoustic stimulation

The acoustic stimuli (pure tone and noise bursts) were generated and delivered to the subjects by using a Tucker-Davis Technologies System 3 (TDT 3, Tucker-Davis Technologies, Alachua, FL, USA). A real-time processor (RP 2.1) and a custom-made program written with RPvdsEx software were used to synthesize the sound signals, the intensities of which were controlled by a programmable attenuator (PA5). The synthesized signals were amplified and delivered by an electrostatic speaker driver (ED1) and a free-field ultrasonic loudspeaker (ES1, frequency range 2–110 kHz). The loudspeaker was calibrated with 1/8 and 1/4 inch microphones (Brüel and Kjaer 4138, 4135, Naerum, Den-mark) and an amplifier (Brüel and Kjaer 2610, Naerum, Denmark) at the beginning of the experiment. The sound parameters were controlled by Brain Ware software running on a computer. The sinusoidal stimulus waveforms started at a zero-phase angle. The sound intensity was expressed as sound pressure level (SPL, 0 dB re 20 μPa).

### Data acquisition

Although AEPs recorded with surface electrodes are easily obtained and can reflect acoustic evoked electrical activities of all auditory pathways^5^, those AEPs have more noise and require a longer time to extract^53^. Therefore, we inserted electrodes into the ICC to record AEPs with less noise. Such deep-brain AEPs only reflect the acoustic evoked electrical activities of auditory pathways around the electrode tip^54^. The prepared mouse was immobilized on the anti-vibration table again and its head was horizontally oriented toward a loudspeaker located 30 cm away. The covers and dura of the exposed brain were removed. By using a microdriver (Narishige MO-10, Japan), glass micropipettes (tip diameter: approximately 1 μm; impedance: 7–12 MΩ; filled with 2 M sodium acetate and 3% pontamine sky blue) were slowly penetrated into the mouse’s ICC, in which the neurons have short response latencies (6–10 ms for noise response), sharply tuned tonal receptive fields as well as a dorsal-to-ventral gradient of characteristic frequency (from low to high)^55^,^56^,^57^. The penetration depth was 100–1800 μm from the surface of the ICC. During penetration, the AEP was detected with a sample rate of 25 kHz by presenting 50 ms noise bursts (60 dB) to the mouse. The electrical signals were amplified 10,000 times, filtered by a band-pass of 10–300 Hz with a digital amplifier RA16 and recorded and displayed with Brain Ware software.

After AEP was detected in the ICC, a frequency scan (F-scan) was performed in which pure tone bursts (from 2 to 64 kHz in 1 kHz steps, 80 dB SPL, 50 ms duration, 5 ms rise-fall time) were randomly presented at a rate of 1/s with 10 repetitions to identify the BF of the recording site (Fig. 1c). Then, the mouse was anesthetized with sodium pentobarbital (60–70 mg/kg i.p.). Once the mouse was immobilized on the table without paw withdrawal (approximately five minutes after pentobarbital injection), an intensity scan (I-scan) was performed and was repeated every 10 min until the mouse limbs autonomously moved and paw withdrawal occurred. In this scan, the frequencies of pure tone bursts (50 ms duration with a 5 ms rise-fall time) were set at BF and were randomly presented from 30 to 90 dB SPL in 10 dB steps at a rate of 1/s and repeated 10 times. The AEPs (time window: 500 ms after stimulus onset) and the acoustic stimuli parameters in a recording session were recorded and stored in a DAM file.

The exposed brain was treated with physiological saline continuously to prevent the tissue from drying, and the pinnae were maintained as in a normal awake mouse during recording. After the recording, pontamine sky blue was iontophoretically (~20 μA, 15 min) applied to the recording site with a microiontophoresis (Neurophore BH-2, Holliston, MA) to confirm the recording location in ICC. Data recorded outside the ICC were discarded.

The above AEP recording was performed without body warming, temperature monitoring and SPO_2_ monitoring for mice. Pentobarbital can induce hypothermia^35^ and hyoxemia^34^. And hypothermia^51^ and hyoxemia^45^ can change AEP. To analyze the effects of hypothermia and hyoxemia on AEP, the rectal temperatures (home-made thermometer) of eleven mice and the SPO_2_ (pulse oximetry, Nellcor, N-550) of five mice were measured every 10 min after pentobarbital injection without body warming. In addition, with body warming (home-made homeothermic blanket), rectal temperature monitoring and SPO_2_ monitoring, AEP was also re-recorded in five mice, as AEP recording in mice without body warming.

Ketamine, urethane and sodium pentobarbital are three common anesthetics for animal experiments. Ketamine is usually mixed with other anesthetics for use^58^. Urethane has a long anesthesia time^59^. Thus, we chose sodium pentobarbital for our experiment, as it can be used alone and has a relatively short anesthesia time (2–3 h, preliminary experimental data). In addition, noises and clicks with a wider frequency range can activate more auditory pathways^60^. Therefore, tones, which activate a relatively simple auditory pathway, were chosen as the acoustic stimuli to reduce data variability.

### Data processing

For off-line data processing, we employed a custom-made Matlab program. First, we extracted and averaged the ten AEPs corresponding to each identical stimulus to measure the latency and amplitude. Then, we plotted the latencies and amplitudes of AEPs against anesthesia time and acoustic intensities. To fit latency-intensity curves, DCASF (for details, see Results) was used. With this fitting method, we obtained latency and intensity shifts (∆*L* and ∆*I,* respectively) of all latency-intensity curves. Then, we plotted ∆*L* and ∆*I* against anesthesia time. Next, the SPO_2_ and temperatures were also plotted against anesthesia time. In addition, the L_80_-, A_80_-, ∆*L*-, ∆*I*- and temperature-time curves were normalized and fit with a polynomial regression equation (polynomial order = 4). After this fitting, five groups of absolute values of residuals were obtained and compared.

The values of relevant parameters were calculated in Microsoft Excel software (version 2003). All statistical analysis was performed in SPSS statistical software (version 13). Before performing appropriate parametric statistics, datasets were first tested for normal distribution (Shapiro-Wilk test) and equal variances (Levene test). Normally distributed data were presented in figures as mean ± SD. Non-normally distributed data were presented in figures as *P*_25_, *P*_50_ , *P*_75_ and mean ± SD. For two-group comparisons, unpaired *t* test (Normally distributed data) or 2 Independent Samples Tests (Non-normally distributed data) was applied to test significance (two-tailed). For three or more group comparisons, one-way ANOVA (Normally distributed data) or K Independent Samples Tests (Non-normally distributed data) was applied to test significance (two-tailed). For one-way ANOVA, LSD’s test was used to further compare group means. For K Independent Samples Tests, 2 Independent Samples Tests was used to further compare any two group data. In most cases, a *p*-value less than 0.05 was deemed significant. However, in multiple comparison 2 Independent Samples Tests, a *p*-value less than 0.008 (0.05/6) was deemed significant. The data fitting and plotting were carried out in OriginPro software (version 8). The figures were arranged in Canvas software (version 9).

## Additional Information

**How to cite this article**: Huang, B. *et al.* Latency of auditory evoked potential monitoring the effects of general anesthetics on nerve fibers and synapses. *Sci. Rep.* **5**, 12730; doi: 10.1038/srep12730 (2015).
